# Effectiveness of resilience training intervention on psychological capital of the underprivileged widowed women of Fasa City, Iran

**DOI:** 10.1186/s12905-022-01886-9

**Published:** 2022-07-21

**Authors:** Abdolrahim Asadollahi, Leila Karimpoor, Mohammad Hossein Kaveh, Leila Ghahremani

**Affiliations:** 1 Research Center for Health Sciences, Institute of Health, Department of Health Promotion and Gerontology, School of Health, Shiraz University of Medical Sciences, Shiraz, Iran; 2 The Middle East Longevity Institute in Abyad Medical Centre, Abdo Center, Abyad Medical Centre, Azmi Street, Tripoli, P.O. Box: 618, Lebanon; 3 Research Center for Health Sciences, Institute of Health, Department of Health Promotion, School of Health, Shiraz University of Medical Sciences, Razi Ave, Shiraz, Iran

**Keywords:** Training, Widowed women, Resilience, Psychological capital

## Abstract

**Introduction:**

Women heads of households (widows or divorcees) are vulnerable groups in society who face various psychological problems and have less resilience than other women. Therefore, the present study was conducted to determine the effectiveness of resilience educational intervention on the psychological capital of poor widows in Fasa city, south Iran.

**Materials and Methods:**

In this quasi-experimental study, 120 widows covered by Imam Khomeini Relief Foundation were selected by a simple random sampling method based on a random Efron algorithm (Efron coin) and randomly assigned into two interventions and control groups (60 people each) in the second half of 2021. About 8 training sessions on resilience and psychological capital were held for the experimental group through training clips, audio transmissions, and podcasts. Data were collected using demographic characteristics forms, widowers' resilience assessment questionnaires, and Luten's psychological capital scale for testing and control before and two months after the intervention. Data were analyzed with Chi-square test, independent and paired t-test using SPSS V. 26 software.

**Results:**

There were differences between the two groups at pretest in demographic variables and psychological capital and its subscales, and resilience and its subscales (*P* ≥ 0.05).. But two months after the educational intervention, a statistically significant increase was observed in the experimental group in these variables compared to the control group (*p* < 0.05). It indicates that there was an improvement in the outcomes from pretest to post-test within the intervention and no change in the outcomes over time within the control group.

**Discussion and Conclusion:**

Using a resilience-based educational approach as a novelty of this research to promote psychological capital and resilience can increase resilience and psychological capital in widows. Then, considering the positive effect of this educational approach and the low cost of this intervention, it seems that the implementation of such interventions should be included in the plans related to widows.

**Supplementary Information:**

The online version contains supplementary material available at 10.1186/s12905-022-01886-9.

## Introduction

Widowed women or women without husbands refer to those who live single for various reasons such as the husband's death, divorce, abandonment, and not marrying afterward [1, 2]. In America, widows amounted to 2.5% men and 9.7 women [3]. According to the census in 1395, nearly 0.7% of men and 5.01% of women are widowed [4]. In Iran, as this group of women does not have the power to manage their lives, their children turn to support organizations. They are covered by the Relief Foundation and State Welfare Organization. In such a way that of nearly 2,500,000 head of household women, 1,100,000 persons are under the coverage of relief foundation, and 180,000 persons are under the coverage of State Welfare Organization [5]. Given that women are living longer than men and are younger than their spouses at the time of marriage, then they form the dominant population of the widowed [4], and the issue of widowhood is more common in women [5]. While still moaning about their missed husbands as to the most critical stressful event in their lives, these women should take the responsibility of the family [6].

Windowed women are subject to behavioral and social consequences resulting from widowhood. These consequences include considerable changes in the friends and social relations in this period (such as the decrease in audiences and social networks); meanwhile, most widows require revising their social roles that this role revising is usually accompanied by the feeling of loneliness which is one of the most challenging aspects of widowhood [7]. Widowed and divorced women are subject to stigma and suffering [8]. Meanwhile, they are also subject to financial threats after their husband's death as their incomes and benefits, and insurance terms may decrease with the death of the husband [9]. Studies confirmed that widowed women have more stress than those having husbands to manage their lives and are faced with the problems such as physical diseases, sleep problems and depression, and early death, which finally affect their well-being and families [2, 10, 11]. Nevertheless, some can cope easier and reflect more flexibility to the deprivation of husband and adjustment with the issue of widowhood which can improve their health and quality of life during the time [11, 12].

Resilience is often considered a favorable psychological structure that affects the perception of the people from well-being [13] and is considered an important and influential factor on the persons’ adjustability in the experience of deprivation and death of the husband [11]. Resilience is the dynamic process of adjustment and positive coping with unpleasant and bitter experiences in life [14, 15], which is not only resistant against damages or threatening conditions and the active and constructive involvement of the person in the environment. Since Resilience is the dynamic process of adjustment and positive coping with unpleasant and bitter experiences in life. Resilient persons have less feeling of loneliness and frustration, show better tolerance against problems, and have excellent function against difficulties and incompatibilities and will be increased by social support [15, 16]. Various studies indicated that resilience improvement could promote mental health, well-being, and psychological; capital in widowed women [6]. Psychological capital is a positive psychological state and a realistic and flexible approach towards life which is defined by four components: hope (will, dynamism and strategy), self-efficiency (self-esteem and belief to the realization of the goals and responsibilities), resilience (restoration, resurrection, going beyond the problems) and optimism (upbeat attribution style regarding the events and positive future expectation). Luthans believes that when these components are synthesized, forming a high-level structure which is called psychological capital with trying to change stressful situations [17–19].

Meanwhile, the results indicated that training improves resilience and its positive effect on the health and well-being of people [20]. However, no study has been conducted regarding the effectiveness of these components on widowed women. Then, given that head of household women (divorced or widowed) are one of the vulnerable strata of society due to social, economic, cultural, and employment situations and are faced with various psychological problems such as daily stress, inability to express emotions and, consequently, physical and mental problems [2], their level of resilience is lower compared to other women [15]. Ignoring this group of women causes several problems, such as pessimism, absurdity and meaninglessness, and dissatisfaction with life. Therefore, low resilience and not using safe coping strategies cause these women to turn to corruption and deviation and bring educational problems for their children and the prevalence of mental disorders throughout society [20].

Additionally, Training as a factor in promoting divergent skills has long been used in various interventions [9]. Numerous educational interventions have been implemented to increase psychological capital and resilience in individual or group programs focusing on their subscales [21]. Most interventions have confirmed the positive effect of education on resilience and psychological capital as well [22]. Then it is necessary to choose and apply practical training and therapeutic interventions in the new vision and novelty to decrease their problems in different to previous studies; therefore, the current study aims to determine the effectiveness of training intervention of resilience on psychological capital of the underprivileged widowed women of Fasa City (the south Iran) in the second half of 2021.

## Methodology

### Population and sample size

This quasi-experimental study was conducted to determine the effectiveness of interventional training of relevance on the psychological capital of underprivileged widowed women who are widow, housholder, and low-income from Fasa city, south iran in 2020. The understudied population was widowed women under the coverage of Imam Khomeini Relief Foundation of Fasa city, south Iran. According to the confidence level of 95%, power 99% and the level of significance 0.05, the design effect size of the study model is 0.83, and with a standardized squared score of 0.33 in similar study [17], the sample size with 10% dropout was 120 widows. Sampling was done by simple random method. At first, the list of widows under the auspices of Imam Khomeini's relief committee in Fasa city (2091 widows) was extracted and defined as the ID number variable in the software and entered into PASS software version 15. Their Random Allocation is based on Efron's Biased Coin algorithm, which is a restrained randomization method; Search iterations were performed by determining one hundred times and 60 people in each group and a total of 120 people were selected. No samples were excluded in the fallow up.

### Inclusion and exclusion criteria

Inclusion criteria for the study were having age above 50 years, reading and writing literacy, at least five years history of widowhood, access to the smartphone, and living in Fasa city. Exclusion criteria of the study include dissatisfaction with participating in the study, being absent more than one session from training classes, and stressful events occurring such as death and lack of family members, accidents, or disease.

### Research design

Firstly, after taking a license from the university's ethical committee and after conducting the required coordination and taking related licenses, a list of widowed women 50 years old and above was provided by the confirmation of the CEO of Relief foundation of Fasa city. Samples were selected based on the inclusion criteria of the study. Necessary explanations regarding the present study were provided by telephone. After taking informed consent, they were included in the study, and questionnaires were submitted to them. Eight training sessions of 60 min were held about resilience and psychological capital for the experimental group. Considering the existence of COVID-19, only one in-person session was held at Relief foundation of the City for introduction, completing the questionnaire, and providing preliminaries. The remaining seven sessions were held virtually using training clips, sending voices, podcasts, and so on. Also, to achieve the total number of employees in each group and attract employees and provide satisfaction, the researcher donated internet charging packages, encouraged older widows to participate, and offered the samples one free mobile SIM card. To observe the principle of educational justice, equal access to health services, and rules of medical ethics, training sessions of the intervention group were held for the control group, too, after the completion of the study (See Additional file 1 for more details).

### Instrumentation

Data collection tools include demographic specifications (age, underlying disease, level of literacy, income independence, number of children, etc.) and a questionnaire to assess widowed women’s resilience and Luthans’s psychological capital scale.West and his colleagues designed the Widowhood Resilience Scale in 2019. This questionnaire includes 12 questions and six subscales (perseverance: items 1 to 6, social support: items 7 to 10, living at present: items 11 to 15, Help-seeking: items 16 to 18, consistency: items 19 to 20, future perspective: items 23 to 25). It has to be answered in five points on the Likert scale (strongly agree, agree, no opinion. disagree, strongly disagree) and scores on this scale vary between 25 and 125 [23]. The Yarelahi and her colleagues’ study in 2022 among Iranian samples has indicated to reliability of this instrument with Cronbach Alpha 0.86, ICC ≥ 0.82, and criterion-related validity was more than 0.75 (Yarelahi et al. in press).

Luthans has designed a psychological capital questionnaire. This questionnaire includes 24 questions and four sub-scales (self-efficacy: items 1 to 6, hope: items 7 to 12, resiliency: items 13 to 18, optimism: items 19 to 24). Each sub-scale includes six items and subject answers to each item in 6 points Likert’s scale (strongly agree to disagree strongly). To obtain psychological capital’s score, first, we gained the scale of each sub-scale separately and the response rate of this study was 100%. We then considered the sum of the total score of psychological capital [24]. Both experimental and control groups completed questionnaires before and two months after intervention in person and by telephone. Also, The Hashemi Nosratabad and his colleagues’ study in 2011 among Iranian samples has indicated to reliability of this instrument with Cronbach Alpha 0.82, ICC ≥ 0.80 [25].

### Ethical consideration

It is worth mentioning that Code has approved this study of Ethics No. IR.SUMS.REC.1399.1024 at Shiraz University of Medical Sciences. To observe ethical considerations in this study, the aims, importance, and necessity of conducting this research plan were explained to the subjects. They were justified, and their consent was obtained. Given the unique social status of widowed women, and it does not create a sense of pity for them, samples were assured that information would be kept confidential.

### Statistical analysis

Data analysis was conducted using SPSS 22 software at a significant level of 0.05. To explain qualitative data, mean, standard deviation, and for qualitative data frequency, percentage, and independent t- statistical test, paired t-test and Chi-Square were applied. First, the normality of quantitative data was examined using the Kolmogorov–Smirnov test (*P* ≥ 0.5). The datasets generated during and/or analysed during this research are available from the corresponding author on reasonable request. Additionally, the pretest data were also considered the baseline for the intervention evaluation. The assumptions for using independent and paired *t*-tests and Chi-square i.e. scale of measurement, random sampling, normality of data distribution, adequacy of sample size, and equality of variance in standard deviation were aobtained in the study.

## Results

The average age of subject women at the time of widowhood was 50.20 ± 2.77 and 50.91 ± 2.95 in experimental and control groups, respectively. According to an independent t-test, no significant difference was seen between the two groups in age (*P* = 0.064) and age at the time of widowhood (*P* = 0.174). 44.66% of the widowed women in the experimental group and 51.66% in the control group were affected by a disease that in both groups, blood pressure, diabetes, and heart diseases have the highest frequency. The categorical variables of demographic information in both control and experimental groups was assessed using the Chi-Square test, and no significant differences were seen between both groups (see Table 1).

**Table 1:** Comparing demographic characteristics of widowed women in both control and experimental groups

Variable	Experimental group		Control group		*P*-value*
	n	%	n	%	
*Education*					
Literacy for reading and writing	5	8.3	4	6.66	0.229
Under diploma	47	78.34	54	90	
Diploma	8	13.33	2	3.34	
Occupation					
Housewife	36	60	39	65	0.706
Employed	24	40	21	35	
Number of children					
*Daughter*					
5 and less	42	70	38	63.34	0.439
6 and more	18	30	22	36.66	
*Son*					
5 and less	28	46.66	30	50	0.715
6 and more	32	53.35	30	50	
*Status of life*					
With children	51	85	55	91.66	0.255
Alone	9	15	5	8.32	
*Duration of widowhood*					
Less than 1 year	6	10	10	16.66	0.185
1–2 years	25	41.66	16	26.67	
3–5 years	29	48.34	34	56.67	

^*^Chi-square statistical test

Before the training intervention, there was no significant difference between the two experimental and control groups in the amount of psychological capital and its sub-scales. But two months after the training intervention, a significant increase was seen in the experimental group regarding the psychological capital and its sub-scales compared to the control group (see Table 2).

**Table 2:** Comparison of psychological capital and its sub-scales in two experimental and control groups, before and 2 months after training intervention

Variable	Groups	Before intervention	After intervention	Student’s *t*-test^a^	Effect size: Cohen’s d	P*
		Mean ± SD	Mean ± SD			
Self-efficiency	Experimental (n = 60)	18.2 ± 1.8	19.1 ± 3.4	0.884	0.64	0.001
	Control (n = 60)	18.2 ± 1.9	18.3 ± 1.9	0.870	0.05	0.184
	Student’s t-test^b^	0.870	0.875	–	–	
	Effect size: Cohen’s d	–	− 0.29	–	–	
	P**	0.962	0.001	–	–	
Optimism	Experimental (n = 60)	21 ± 1.05	23.7 ± 1.5	0.856	0.81	0.001
	Control (n = 60)	21.1 ± 2.3	21.4 ± 1.7	0.842	0.15	0.167
	Student’s *t*-test^b^	0.590	0.687	–	–	
	Effect size: Cohen’s d		− 1.84	–	–	
	P**	0.973	0.001	–	–	
*Psychological capital*						
Hope	Experimental (n = 60)	18.1 ± 2.1	25.6 ± 2.7	0.870	0.98	0.001
	Control (n = 60)	18.03 ± 2.1	17.7 ± 2.3	0.856	− 0.19	0.132
	Student’s *t*-test^b^	0.420	0.570	–	–	
	Effect size: Cohen’s d	–	-2.83	–	–	
	P**	0.132	0.001	–	–	
Resilience	Experimental (n = 60)	20.3 ± 2.1	25.4 ± 2.07	0.620	2.44	0.001
	Control (n = 60)	20.4 ± 1.4	20.5 ± 1.9	0.491	0.06	0.145
	Student’s *t*-test^b^	0.684	0.687	–	–	
	Effect size: Cohen’s d	–	− 2.46	–	–	
	P**	0.145	0.001	–	–	
Total	Experimental (n = 60)	77.3 ± 6.6	81.9 ± 7.8	0.856	0.63	0.001
	Control (n = 60)	77.9 ± 5.6	78.3 ± 5.7	0.842	0.07	0.159
	Student’s *t*-test^b^	0.772	0.978	–	–	
	Effect size: Cohen’s d	–	-0.52	–	–	
	P**	0.159	0.001	–	–	

^a^Independent student *t*-test

^b^Paired student *t*-test, DF ≥ 51

According to the Table 2, Cohen's d as effect size coefficient indicates the high effectiveness of the intervention in this study, which had a higher effect coefficient belonging to resilience of Psychological Capital among older samples (Cohen’s d = 2.44).

As Table 3 shows two months after the training intervention, a significant increase was seen in the experimental group regarding the resilience outcomes and its sub-scales compared to the control group (Table 3).

**Table 3:** Comparison of the resilience and its sub-scales in two experimental and control groups, before and 2 months after training intervention

Variable	Groups	Before intervention	After intervention	Student’s t-test^a^	Effect Size: Cohen’s d	P*
		Mean ± SD	Mean ± SD			
Perseverance	Experimental (n = 60)	17.3 ± 1.8	23.3 ± 1.3	2.35	3.82	0.001
	Control (n = 60)	17.8 ± 1.9	17.4 ± 1.8	2.78	− 0.21	0.262
	Student’s *t*-test^b^	3.45	4.02	–	–	
	Effect size: Cohen’s d	–	− 3.75	–	–	
	P**	0.001	0.262	–	–	
Social support	Experimental (n = 60)	10.8 ± 1.7	15.8 ± 0.8	1.281	3.76	0.001
	Control (n = 60)	11 ± 1.7	10.7 ± 1.7	1.305	− 0.17	0.223
	Student’s *t*-test^b^	2.65	2.48	–	–	
	Effect size: Cohen’s d	–	3.84	–	–	
	P**	0.001	0.224	–	–	
*Resilience*						
Living in present	Experimental (n = 60)	15.5 ± 1.4	19.6 ± 1.3	1.442	3.1	0.001
	Control (n = 60)	18.8 ± 1.6	15.4 ± 1.4	1.435	− 2.26	0.263
	Student’s t-test^b^	1.457	1.414	–	–	
	Effect Size: Cohen’s d	–	− 3.11	–	–	
	P**	0.001	0.263	–	–	
Help-seeking	Experimental (n = 60)	9.1 ± 1.3	12.6 ± 0.8	1.442	3.24	0.001
	Control (n = 60)	9.1 ± 1.3	9.4 ± 1.4	1.435	0.22	0.961
	Student’s t-test^b^	1.571	1.171	–	–	
	Effect Size: Cohen’s d	–	− 2.81	–	–	
	P**	0.001	0.972	–	–	
Consistency	Experimental (n = 60)	9.1 ± 1.9	14.9 ± 1.2	1.428	3.65	0.001
	Control (n = 60)	9.3 ± 2.03	9.08 ± 1.9	1.421	− 0.12	0.972
	Student’s t-test^b^	1.414	1.507	–	–	
	Effect Size: Cohen’s d	–	− 3.66	–	–	
	P**	0.001	0.954	–	–	
Future perspective	Experimental (n = 60)	10.1 ± 1.2	12.3 ± 0.8	1.171	2.16	0.001
	Control (n = 60)	11.1 ± 1.2	10.9 ± 1.1	1.297		0.942
	Student’s *t*-test^b^	1.283	1.671	–	–	
	Effect size: Cohen’s d	–	− 0.17	–	–	
	P**	0.001	0.532	–	–	
Total	Experimental (n = 60)	72.05 ± 4.1	98.7 ± 4.3	2.251	6.24	0.001
	Control (n = 60)	72.7 ± 4.01	72.8 ± 4.02	1.260	0.02	0.155
	Student’s *t*-test^b^	2.281	2.281	–	–	
	Effect size: Cohen’s d	–	− 6.43	–	–	
	P**	0.001	0.537	–	–	

^a^Independent student *t*-test

^b^Paired student *t*-test, *DF* ≥ 48

According to the Table 3, Cohen's d as effect size coefficient indicates the high effectiveness of the intervention in this study, which had a higher effect coefficient belonging to total score of Resilience among older widows (Cohen’s d = 6.24).

## Discussion

The current study assessed the effectiveness of a training approach based on resilience in the widowed women covered by the Imam Khomeini Relief Foundation of Fasa city. This study indicated that experimental and control groups did not significantly differ from the demographic variables’ perspectives. Lack of difference between the study groups from demographic variables’ perspective indicated that stages of the study, including sampling, were conducted with high and appropriate accuracy, and the confounding effect and demographic variables were controlled. Therefore, attribution of observed changes in the experimental group is empowered.

The current study results indicated that two months after the intervention, the average score of psychological capital and its sub-scales (hope, resilience, optimism, and self-efficiency) increased significantly in the experimental group compared to the control group. This finding supports the implementation of resilience training interventions to promote psychological capital in widowed women. By increasing psychological capital, the individual can delineate new goals and try to achieve her goals with higher motivation, making more optimistic attributions about herself, experiences, and the future. The person can generate more favorable judgments about herself and her abilities and increase her resilience in facing difficulties and misfortunes with more strict feedback and strategies under stressful situations [26]. Promoting psychological capital in the increasing population of widowed women can help this group experience a healthier and happier life [27]. Studies by Vahidian et al. [28] and Vale et al. [29] reported increased psychological capital after the training intervention, respectively, in the group of female nurses and female teachers, which is congruent with the current study.

Align with the current study, the study results of Chesak et al. [30] indicated that resilience-based intervention has effectively promoted cases such as hope, happiness, and resilience. Although contrary to the current study, the target group of this study was not widowed women and did not consider specifically psychological capital. It confirms the positive effect of resilience training intervention. People who experience high hope experience lower negative emotional reactions than people with low hope. One reason is that people with high hope compared to those having low hope have the highest capacity to find alternative paths toward their initial goals [31]; with the increased cognitive resilience flexibility raised in the individual, her persistence increases. Instead of inactive confrontation in facing stressful events, uses more active, concentrated, and task-oriented confrontations [32].

The current study results in optimism and self-efficiency sub-scales are consistent with the study of Molhtari et al. [33]. By applying effective coping strategies such as reassessment and problem-solving, Optimistic individuals adjust better to mental pressures [34]. Furthermore, by increasing self-efficiency, the confidence of the person in her abilities increases, fear of failure decreases in her, and this thinking grows in the person that she can control the events that affect her life [35] Diagan et al. indicated the positive role of psychological capital in the empowerment of women [36]. Studying various studies and the current study results indicated that the promotion of psychological capital could empower widowed women who are vulnerable groups in different dimensions of life [37]. According to the results of this study and its comparison with other studies, it can be concluded that training intervention based on resilience can be effective in the improvement of some skills such as self-efficiency and cases such as well-being and the quality of life. What differentiates this study from others is the application of resilience-based training to improve psychological capital in widowed women.

The current study results indicated that after two months of intervention, the average scores of resilience and its subscales (perseverance, social support, leaving in the present, help-seeking, future perspective) had increased significantly in the experimental group compared to the control group, which indicated the effectiveness of training intervention on the improvement of the resilience in widowed women.

The results of the present study indicated that two months after the intervention, the average score of resilience and its sub-scale (perseverance, social support, living in the present, help-seeking, future perspective) increased significantly in the experimental group compared to the control group, which indicated the effectiveness of the training intervention on the improvement of resilience of the widowed women. The study of Mahdavian far et al. [38] conducted a group intervention to improve resilience in the divorced head of household women. This study, likewise, the results of the current study indicated that training intervention has been effective in promoting resilience in women's heads of house. The difference between this study and the current study is that the target group of this intervention was divorced women. The intervention was done as group therapy, while in the present study, widowed women were under the group training intervention. Asad O Lah Toisarkani et al. [39] conducted two training interventions for the mother students to improve their resilience. The type of training intervention applied in this study had no significant effect on the resilience of the target group.

Meanwhile, unlike the current study, mothers under the intervention were not widowed. Likewise, the current studies, Mahaffey et al. [40] and Yoosefi Lebni et al. y[41] arranged and conducted a training intervention based on resilience. Unlike the current study, the target group of this study, which was widowed women, was health workers in disasters. However, the study results to resilience-based training intervention can effectively promote some behaviors of a healthy lifestyle, which can be known to align with the results of the current study.

## Conclusion

Totally, the results of the study indicated that using resilience-based training intervention for the promotion of psychological capital and resilience could improve resilience and psychological capital in widowed women. Therefore, given the positive effects of this training approach and the low cost of this intervention, it seems better to include in the planning related to the widowed women. The centeral message is that Resilience-based educational approach to promote psychological capital and resilience, could lead to increase resilience and psychological capital in widows. Therefore, considering the positive impact of this educational approach and the low cost of this type of intervention, it seems that it is better to include the implementation of such interventions in the health policy planning of widows’ everyday's life.

## Limitations and recommendations

A limitation of this study is that it has been done virtually among widowed women covered by Imam Khomeini Relief Foundation of Fasa City in south Iran due to the holding of distance learning courses in all non-profit and public NGOs across the country. Also, widowed women with cognitive and somatic disorders were excluded from the study. The findings cannot be generalized to all widowed women. On the other hand, considering the COVID-19 pandemic, it was required to hold the training courses virtually, which may change the study results compared to holding the sessions in person. One of the strengths of this study is the concurrent attention to two components of resilience and psychological capital, which has received less attention in other studies and using resilience-based training intervention on the improvement of psychological capital in widowed women.

## Supplementary Information

**Additional file 1. Supplementary Table 1.** Study Protecol and Content of training sessions for widowed women in the experimental group.

## Data Availability

The datasets used and analyzed during the current study are available from the corresponding author on request. The data are not publicly available due to privacy or ethical restrictions.
