# Supplementary material for: Effectiveness of resilience training intervention on psychological capital of the underprivileged widowed women of Fasa City, Iran
Source: BMC Womens Health. 2022 Jul 21;22:302. doi: 10.1186/s12905-022-01886-9 (PMC9302561; doi:10.1186/s12905-022-01886-9)
Supplement: Supplementary file 1 — Additional file 1. Supplementary Table 1. Study Protecol and Content of training sessions for widowed women in the experimental group. [file 12905_2022_1886_MOESM1_ESM.doc]

Supplementary Table 1- Study Protecol and Content of training sessions for widowed women in the experimental group

| **Session** | **Time** | **The subject of in-person and online sessions** | **Object** | **Method of holding** |
| --- | --- | --- | --- | --- |
| first | 60 min | Communication and introduction- introducing the plan and title of the study- explaining the intervention | Breaking the ice of communication- explaining the intervention | In-person and formation of virtual group |
| Second | 60min | Resilience | Explaining resilience and the ways to improve it |  |
| Third | 60 min | Psychological capital- Reviewing previous sessions- 15 min | Explaining the psychological capital and its dimensions | Training clip pamphlet, voice |
| Forth | 60 min | Optimism- Reviewing previous sessions- 15 min | Explaining optimism and the ways to improve it | Training clip |
| Fifth | 60 min | Hope- Reviewing previous sessions- 15 min | Explaining about the hope and the ways to improve it | Training clip- voice |
| Sixth | 60 min | Self-efficient, Reviewing previous sessions- 15 min | Explaining about the Self-efficient and the ways to improve it | Training clip- voice |
| Seventh | 60 min | Problem-solving skill- Reviewing previous sessions- 15 min | Training problem-solving skills and communicative skills | Training clip |
| Eighth | 60 min | Conclusion | Final conclusion- taking feedback |  |
